# Mesalamine-induced Myocarditis in a Young Athlete: Can He Run Again?

**DOI:** 10.7759/cureus.3978

**Published:** 2019-01-29

**Authors:** Amira Ibrahim, Charl Khalil, Michael Megaly, Mariam Tawadros, Wassim Mosleh, John Corbelli

**Affiliations:** 1 Internal Medicine, State University of New York at Buffalo, Buffalo, USA; 2 Cardiology, Minneapolis Heart Institute and Hennepin Healthcare, Minneapolis, USA; 3 Internal Medicine, Ain-Shams University Hospitals, Cairo, EGY; 4 Cardiology, University of Connecticut Health Center, Farmington, USA; 5 Cardiology, State University of New York at Buffalo, Buffalo, USA

**Keywords:** inflammatory bowel disease (ibd), mesalamine, myocarditis, crohns disease, chest pain

## Abstract

Mesalamine is often used in the treatment of inflammatory bowel disease (IBD). Mesalamine-induced cardiotoxicity has been reported in the literature and is a rare entity. The mechanism of cardiotoxicity remains unclear, however, it is believed to be due to a humoral-mediated hypersensitivity reaction. Patients with mesalamine-induced cardiotoxicity could present with a wide range of cardiovascular symptoms ranging from mild chest pain and shortness of breath (SOB) to cardiogenic shock secondary to left ventricular systolic dysfunction. Symptoms could be associated with elevation in cardiac biomarkers and electrocardiogram (EKG) changes including ST-segment or T-wave abnormalities. We report a case of mesalamine-induced myocarditis in a young athlete presenting with chest pain 10 days after mesalamine therapy was initiated for recently diagnosed Crohn’s disease. Workup was significant for elevated cardiac biomarkers. The diagnosis was confirmed with cardiovascular magnetic resonance imaging (CMR). Immediate cessation of the medication resulted in resolution of symptomatology and normalization of cardiac biomarkers over a 48-hour period. Mesalamine-induced cardiotoxicity is a rare, yet serious side effect that necessitates medical community awareness. CMR is the confirmatory diagnostic modality of choice.

## Introduction

Mesalamine containing products are often used in the treatment of inflammatory bowel disease (IBD) [1]. Cardiac involvement could be an extra-intestinal manifestation of IBD or a medication side effect. Few cases in the literature have reported cardiotoxicity as a rare possible side effect of mesalamine [2-8]. Patients can present with a wide range of cardiovascular symptoms, ranging from mild chest pain and shortness of breath (SOB) to cardiogenic shock secondary to left ventricular systolic dysfunction [2].

## Case presentation

We present a case of a 21-year-old college football player with a medical history of recently diagnosed Crohn’s disease, for which he was started on mesalamine daily, four weeks before his emergency department (ED) presentation. The patient presented to the ED with recurrent intermittent episodes of chest pain over a 24-hour period. He described the chest pain as sharp, sub-sternal pain, 8/10 in severity that started while he was at rest. He experienced two self-resolving episodes; each lasted for an hour before he encountered a third more prolonged episode prompting him to present to the ED. The patient denied having any shortness of breath, cough, fever, runny nose, watery eyes, or other systemic symptoms before his chest pain. The patient had no cardiovascular risk factors and no family history of heart disease.

**Figure 1:**
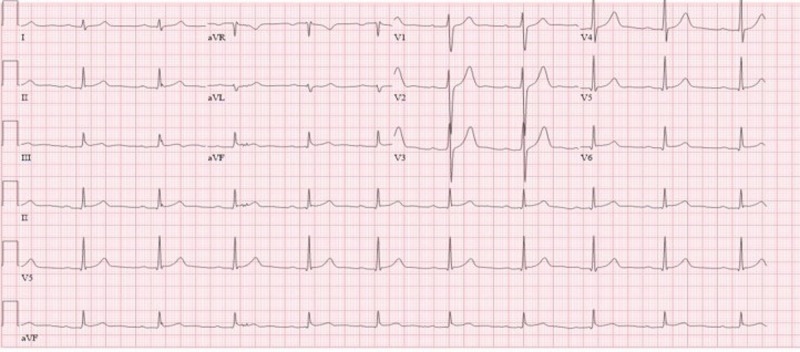
Electrocardiogram (EKG). EKG showing normal sinus rhythm with first-degree heart block and non-specific ST-T changes.

**Figure 2:**
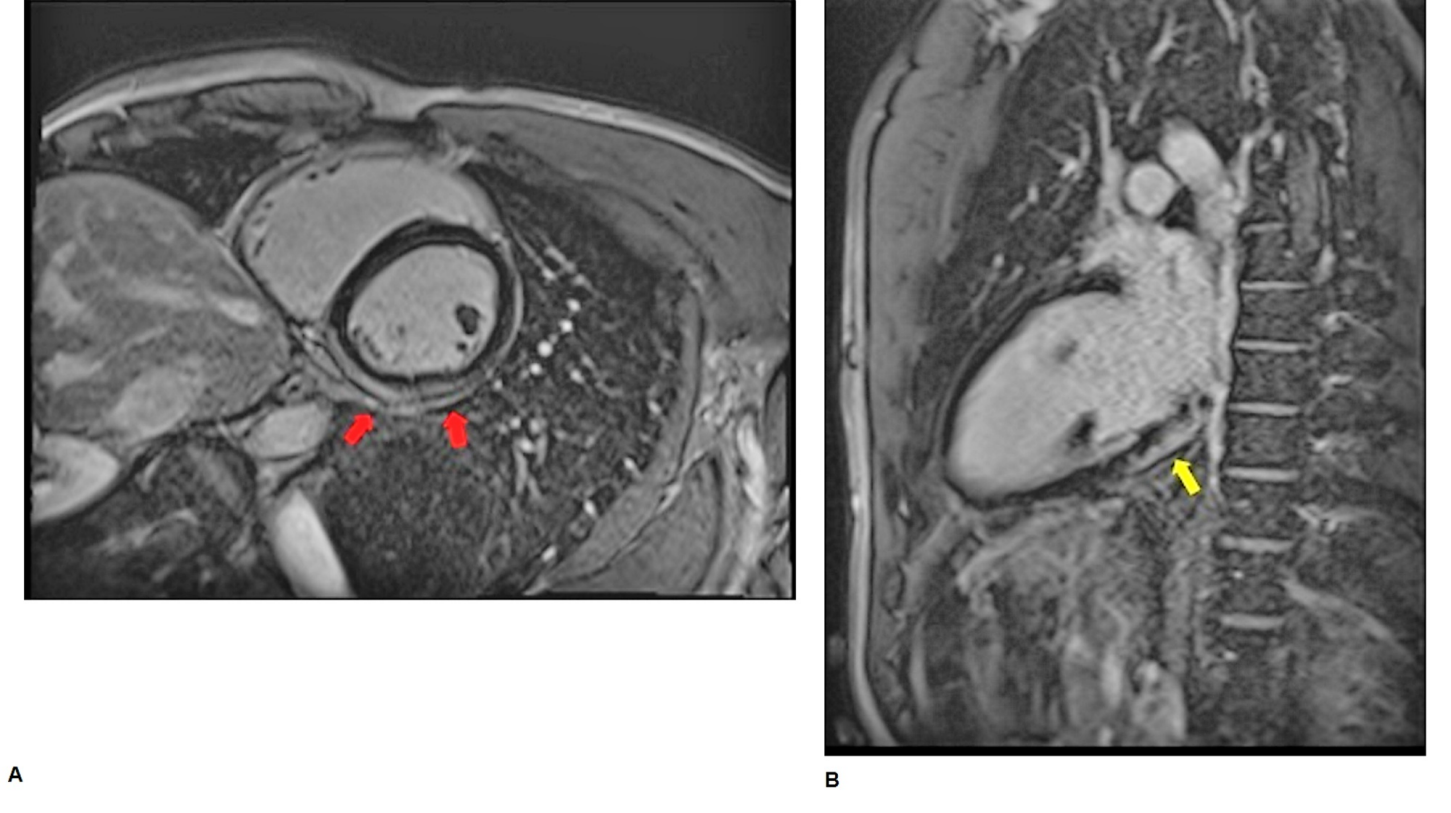
Cardiac magnetic resonance imaging (CMR). A: CMR, axial plane, showing sub-epicardial to mid-myocardial delayed hyper-enhancement involving the basal to mid-inferior and infero-lateral walls consistent with myocarditis (red arrows). B: CMR, coronal plane, showing sub-epicardial to mid-myocardial delayed gadolinium hyper-enhancement involving the basal to mid-inferior and infero-lateral walls consistent with myocarditis (yellow arrow).

The electrocardiogram (EKG) demonstrated normal sinus rhythm with first-degree heart block and non-specific ST-T changes (Figure 1). Cardiac biomarkers were elevated (Troponin I: 2.215 ng/ml and CK: 220 IU/L). The echocardiogram demonstrated normal wall motion and an ejection fraction of 55-60%. The patient’s presentation and elevated biomarkers raised the suspicion for mesalamine-induced myocarditis. A cardiac magnetic resonance (CMR) study was performed and demonstrated subepicardial to mid-myocardial delayed gadolinium hyper-enhancement and edema involving the basal inferior to inferolateral wall, which is a non-ischemic pattern that is consistent with myocarditis (Figure 2). Mesalamine was then discontinued, with subsequent resolution of patient’s chest pain and normalization of troponin levels over a 48-hour period.

## Discussion

Mesalamine is a 5-aminosalicylic acid (5-ASA) containing medication commonly used for the treatment of IBD. Side effects more commonly associated with this medication include headache, fatigue, nausea and abdominal discomfort. The mechanism of mesalamine in the treatment of IBD is not fully understood. It was hypothesized that it interferes with the signaling pathway of γ-form of peroxisomal proliferator-activated receptors and the cyclooxygenase pathway and consequently decreases inflammation in the colon [1]. Mesalamine-induced cardiotoxicity has been reported in the literature and is a rare entity. Patients with mesalamine-induced cardiotoxicity can present with dyspnea, substernal chest pain, fever, leucocytosis, elevation in cardiac biomarkers, ST-segment and T-wave abnormalities on EKG and left ventricular systolic dysfunction [2]. The mechanism of mesalamine-induced cardiovascular toxicity remains unclear. Hypotheses include a humoral-mediated hypersensitivity reaction, direct toxic effect, or an allergic reaction mediated by immunoglobulin E. The humoral-mediated hypersensitivity resulting from antibodies' cross-reactivity between mesalamine and cardiac tissue, seems to be the predominant theory [9]. This latter theory could explain the reason that symptoms are dose independent and can start early during the course of the treatment.

Cardiac involvement in IBD could be an extraintestinal manifestation of the disease or an adverse reaction to a medication [3]. The mesalamine-induced onset of symptoms supports cardiotoxicity within a short period after medication initiation and the resolution of symptomatology upon medication cessation [4]. Most mesalamine-induced cardiovascular toxicity cases occurred 2-4 weeks after treatment was initiated, although it could be delayed in cases of concomitant steroid use [4]. In most cases, symptoms resolved within one week of medication discontinuation [1]. Cardiac involvement as an extra-intestinal manifestation of IBD has a very low incidence. In those cases, cardiac symptomatology can be present early during the course of the disease or manifest years after diagnosis. Thus, it remains a challenge to determine whether cardiac involvement is because of underlying IBD or an adverse effect of this medication. Medication discontinuation and monitoring for resolution of symptoms is a reasonable diagnostic strategy to differentiate between both conditions [9].

Our patient had no symptoms suggestive of viral illness and his symptoms manifested 10 days after he was started on mesalamine. CMR showed subepicardial delayed gadolinium enhancement consistent with myocarditis. His symptomatology resolved and cardiac biomarkers normalized upon discontinuing the medication, which makes mesalamine the most likely cause of his myocarditis.

The use of CMR to confirm the diagnosis of mesalamine-induced cardiotoxicity has been seldom reported [2,5]. Given the potential serious outcomes associated with undiagnosed mesalamine-induced cardiotoxicity, it is essential to acknowledge the presence of such an entity and emphasize that CMR is the diagnostic test of choice.

## Conclusions

Mesalamine-induced cardiotoxicity is a rare, yet potentially a lethal drug side effect that necessitates a high index of suspicion. Patients on mesalamine who present with cardiovascular complaints should have a workup that includes troponin levels, an EKG and early consideration for obtaining a CMR as the confirmatory diagnostic modality of choice. Side effects commonly resolve early in the time course following stopping of the drug.
